# Identification of Cancer Hallmarks Based on the Gene Co-expression Networks of Seven Cancers

**DOI:** 10.3389/fgene.2019.00099

**Published:** 2019-02-19

**Authors:** Ling-Hao Yu, Qin-Wei Huang, Xiong-Hui Zhou

**Affiliations:** ^1^College of Science, Huazhong Agricultural University, Wuhan, China; ^2^Hubei Key Laboratory of Agricultural Bioinformatics, College of Informatics, Huazhong Agricultural University, Wuhan, China

**Keywords:** cancer hallmarks, gene co-expression network, cancer prognosis, pan-cancer analysis, drug target

## Abstract

Identifying the hallmarks of cancer is essential for cancer research, and the genes involved in cancer hallmarks are likely to be cancer drivers. However, there is no appropriate method in the current literature for identifying genetic cancer hallmarks, especially considering the interrelationships among the genes. Here, we hypothesized that “dense clusters” (or “communities”) in the gene co-expression networks of cancer patients may represent functional units regarding cancer formation and progression, and the communities present in the co-expression networks of multiple types of cancer may be cancer hallmarks. Consequently, we mined the conserved communities in the gene co-expression networks of seven cancers in order to identify candidate hallmarks. Functional annotation of the communities showed that they were mainly related to immune response, the cell cycle and the biological processes that maintain basic cellular functions. Survival analysis using the genes involved in the conserved communities verified that two of these hallmarks could predict the survival risks of cancer patients in multiple types of cancer. Furthermore, the genes involved in these hallmarks, one of which was related to the cell cycle, could be useful in screening for cancer drugs.

## Introduction

Cancer is a complex disease characterized by uncontrolled cell growth. The burden of cancer worldwide will increase from 14 million in 2012 to 24 million in 2035 (Stewart et al., 2016) and cancer is one of the leading causes of death in the world (Jemal et al., 2010). Meanwhile, accurate prognosis is essential for the treatment of cancer patients (Domany, 2014). With the development of high-throughput technology, using high-throughput data to screen for genes related to cancer prognosis has become a key technique (Eytan, 2014) and prognostic genes are candidate drug targets. However, the cancer-related genes identified in this way lack robustness (Chang et al., 2005). As different cancers share common characteristics (cancer hallmarks) (Hanahan and Weinberg, 2011), it is of great significance to study the common characteristics of patients with different types of cancer. The genes involved in cancer hallmarks may be more robust.

Recently, several pan-cancer analysis methods have been proposed to identify the common genetic characteristics among multiple types of cancer (Aran et al., 2015; Knijnenburg et al., 2015; Dhawan et al., 2017). However, cancer is a complex polygenic disease (Bredberg, 2011). The occurrence of cancer is usually caused by a combination of genes, while most of the above-mentioned pan-cancer analysis methods ignore the interrelationships among the genes. The biological networks have been proved to be a powerful tool to study complex diseases (Chen et al., 2016a,b, 2017, 2018a,b,c,d,e; Chen and Huang, 2017; You et al., 2017), drug-disease associations (Zhang et al., 2018d,e), drug-drug interactions (Zhang et al., 2017a, 2018a), drug side effects (Zhang et al., 2017b, 2018b), and lncRNA-proteins interactions (Zhang et al., 2018c,f), and thus the adoption of biological networks in pan-cancer analysis may be useful. Conserved functions and genes can be found by analyzing gene co-expression networks of different types of cancer (Yang et al., 2014). Therefore, mining the hallmarks of multiple cancers using gene co-expression networks is a promising approach.

In biological networks, the dense subnetworks that are closely interconnected can work together as functional “communities” (Zhou et al., 2014). The communities in multiple different cancer networks may be essential in these cancers, and thus are more likely to be cancer hallmarks. In this study, we integrated the mRNA expression data of seven types of cancer [ovarian cancer (OV), breast cancer (BRCA), lung adenocarcinoma (LUAD), acute myeloid leukemia (LAML), lung squamous cell carcinoma (LUSC), pleomorphic glioblastoma (GBM), and kidney renal clear cell carcinoma (KIRC)] from The Cancer Genome Atlas (TCGA). First, considering the relationships among genes, we constructed the gene co-expression networks of the seven cancers. Then, using permutation tests, we mined dense clusters (communities) that were conserved in all the networks. Next, we functionally annotated these communities to see whether the genes involved in these communities could reveal the biological mechanisms underlying cancer. Afterward, survival analysis was used to select the communities that could significantly distinguish between cancer patients in terms of survival regarding multiple types of cancer. We regarded the communities that were related to cancer prognosis in multiple types of cancer as cancer hallmarks. Finally, we explored whether the genes involved in the hallmarks could be useful in screening for cancer drugs.

## Materials and Methods

### Data Sets

We downloaded TCGA data sets for nine cancers: OV, BRCA, LAML, LUAD, LUSC, GBM, KIRC, KIPAN, and COAD (Mclendon et al., 2008). The data sets included clinical data (survival time, survival state) and gene expression data (UNC Agilent G4502A_07, level 3). The first seven data sets were used to identified the hallmarks and the last two data sets were used to validate our method. The details are shown in Table 1.

**Table 1:** Details of the cancer data sets.

	Number of patients with	Number of patients with
Data sets	mRNA expression data	clinical data
BRCA	593	528
GBM	473	468
KIRC	72	72
LAML	197	186
LUAD	32	32
LUSC	155	152
OV	559	538
KIPAN	88	88
COAD	172	159

Additionally, drugs, indications, and drug targets were obtained from the Therapeutic Target Database (TTD) (Chen et al., 2002), Drug-Gene Interaction Database (DGIdb) (Wagner et al., 2016), and DrugBank (Wishart et al., 2008; Law et al., 2014). The targets for each drug were set as the targets in any of the three databases.

### Construction of Gene Co-expression Networks

Genes in one functional pathway may be strongly mutually co-expressed, while genes in another functional pathway may be weakly co-expressed (Ruan et al., 2010), and a value-based method may ignore the weakly co-expressed, but essential, gene pairs. Therefore, we applied a rank-based method (Ruan et al., 2010) to construct the gene co-expression network for each cancer type.

First, Pearson correlation (Benesty et al., 2009) was used to calculate the correlation coefficient between each pair of genes based on the mRNA expression data. The calculation method was as follows:

(1) $$r=\frac{1}{n-1}\sum_{\text{i}=1}^{\text{n}}\left(\frac{X_{\text{i}}-\bar{X}}{{\text{σ}}_{\text{x}}}\right)\left(\frac{Y_{\text{i}}-\bar{Y}}{{\text{σ}}_{\text{y}}}\right)$$

where *n* is the number of cancer samples. *X*_i_,*X*, and σ_X_ represent the gene expression value of gene *X* in the i-th sample, the mean value of gene *X* in all the samples, and the standard deviation of gene *X* in all the samples, respectively. In the same way, *Y*_i_, *Y*, and σ_Y_ represent the same meanings of the other gene, gene *Y*, in the gene pair.

Second, for each gene, the top *n* most relevant genes, defined by the correlation coefficient, were selected as its “neighbors.” In this study, adopting a similar strategy to that used in our previous study (Hu and Zhou, 2017), *n* was set at 10.

Finally, all the selected gene pairs were taken to represent the gene co-expression network.

### Network Visualization and Community Detection

We used Cytoscape 3.4.0 to visualize and analyze each co-expression network, and we used the MCODE (Bader and Hogue, 2003) plug-in for Cytoscape to detect the dense clusters (termed communities) in the network.

### Hallmark Mining Based on Permutation Tests

The communities present in all cancers may be cancer hallmarks. Therefore, we used permutation tests to mine the conserved communities present in the gene co-expression networks of all the cancers.

First, the communities in the gene co-expression network of OV were detected by MCODE (Bader and Hogue, 2003).

Second, for the set of genes in each OV community, a permutation test was used to assess whether the number of interactions among the same set of genes in another cancer (cancer *B*) was significantly greater than the number of interactions in a random gene set. The permutation test was performed as follows:

(1) In cancer *B*, the number of edges in the gene set, which involved the same nodes (genes) as in the OV community, was calculated.
(2) The same number of genes was randomly selected, and the number of the edges in the random gene set was calculated.
(3) Step 2 was repeated 1000 times, and the number of random gene sets with no less edges than in the community in question in cancer *B* was counted.
(4) The significance level (*p*-value) of the community in cancer *B* can be calculated as follows:

(2) $$P-\text{value}=\frac{\text{X}}{1000}$$

where *X* represents the number of random gene sets with no less edges than in the community in cancer *B*.

Finally, if the community, which had no less than 5 nodes (genes), was significant (*p*-value < 0.05) in all the cancers, the community was set as a candidate hallmark.

### Survival Analysis of Cancer Patients Using Hallmarks

To verify whether the genes involved in each conserved community could distinguish the prognostic risks of cancer patients, we performed a survival analysis in each cancer data set using the genes in each conserved community as prognostic markers. First, we divided the cancer patients in each data set into two equal groups, with an equal ratio of surviving patients in both. One was the training set and the other was the test set.

Based on the *training* set data, the Cox coefficient of each gene was calculated, which represents the correlation coefficient between the gene expression levels and the prognostic risks of the cancer patients in terms of survival. The Cox coefficient was calculated by the Cox proportional hazards regression (Mauger et al., 2010), which is a semiparametric method and can adjust survival rate estimation to quantify the effect of predictor variables. Here, we applied it to quantify the effect of each gene to the prognostic risks of cancer patients.

Thereafter, we used the Gene expression Grade Index (GGI) formula (Sotiriou et al., 2006) to calculate the prognostic risk in terms of survival of each patient in the test set:

(3) $$\text{GGI Risk Score =}\sum x_{\text{i}}-\sum y_{\text{j}}$$

where x_i_ is the expression level of a gene with a positive Cox coefficient and y_i_ is the expression level of a gene with a negative Cox coefficient.

According to these GGI Risk Scores, the patients in the *test* set were divided into two groups: the patients with a top 50% GGI Risk Score were in the high-risk group and the other half were in the low-risk group. Finally, the log rank test was performed to test whether the difference in the actual survival risk (based on the hazard ratio) between the two groups was significant. The hazard ratio (HR) was used to evaluate the ratio of the hazard rates of the true prognostic risks of the patients in the two groups, which is divided based on the gene expression levels of all the genes in the conserved community.

### Gene Set Enrichment Analysis (GSEA)

We used GSEA (Subramanian et al., 2005) to investigate the biological functions [Gene Ontology (GO): Biological process (BP)] of the candidate hallmarks.

The hypergeometric distribution test was used to test whether the intersection of genes involved in a hallmark *and* genes that were drug targets was significantly greater than the number that would be expected based on chance alone. The *p*-value was calculated as follows:

(4) $$\begin{array}{c} p-value=1-F(x-1/M,K,N)\\ =1-\sum_{i=0}^{\text{x}-1}\frac{\left(C_{K}^{i}\times C_{M-K}^{N-i}\right)}{C_{M}^{N}} \end{array}$$

where *x* is the number of genes in the intersection set, *K* is the number of genes involved in the hallmark, *N* is the number of genes that were drug targets, and *M* is the number of genes overall.

In addition, we used the hypergeometric distribution test to assess whether the proportion of cancer drugs among the drugs selected by screening according to a hallmark was significantly higher than the proportion of cancer drugs among all the drugs in the three databases.

## Results

In this study, we employed a rank-based method to construct a gene co-expression network for each of the seven cancers. Subsequently, power-law fitting was performed to test whether the networks were scale-free. After that, permutation tests were used to mine the conserved communities present in all the networks. In addition, the biological functions of these communities were investigated using an enrichment analysis. Afterward, survival analysis was used to select the communities that could significantly distinguish between cancer patients in terms of survival regarding multiple types of cancer. Finally, the hallmark genes were used to screen for drugs based on the drug targets. The detailed results of our study are as follows.

### Gene Co-expression Networks Are Scale-Free

As the rank-based method (Ruan et al., 2010) can capture both strong mutual co-expression and weak co-expression, we used it to construct a co-expression network for each cancer based on gene expression data from the TCGA. In each network, for each gene, the top 10 most related genes were selected as its “neighbors.” The number of nodes and edges of the gene co-expression networks of all the cancers are listed in Supplementary Table S1, and all the co-expression networks of the seven cancers are shown in Supplementary Tables S2–S8.

Furthermore, power-law fitting was applied to all the networks and the results are shown in Table 2. This table indicates that almost all the correlations and *R*^2^ values of the fitting were >0.80 (except the correlation of the fitting in the LAML network), which indicates that all the networks fitted the power-law distribution exactly. That is, all the gene co-expression networks were scale-free, which conforms with the topological characteristics of biological networks. Therefore, we were able to use the topological components of our networks, such as the communities, for further study.

**Table 2:** Correlations and R-squares of the power-law fitting in the seven networks.

Networks	Correlation	*R*-square
BRCA	0.971	0.948
GBM	0.964	0.958
KIRC	0.839	0.951
LAML	0.443	0.885
LUAD	0.997	0.868
LUSC	0.824	0.952
OV	0.898	0.942

### Functions of the Conserved Communities Are Related to Known Cancer Hallmarks

The hypothesis of this study is that the communities present in multiple gene co-expression networks may be candidate hallmarks. As the OV data set had the largest number of samples, with gene expression data as well as clinical information, the co-expression network of OV was applied to mine the communities using MCODE (Bader and Hogue, 2003). As a result, 450 communities were detected using default parameters. Subsequently, permutation tests were used to verify whether these communities were conserved in the co-expression networks of other cancers. Consequently, 58 communities, which existed in all seven networks, were selected as candidate hallmarks.

**FIGURE 1:**
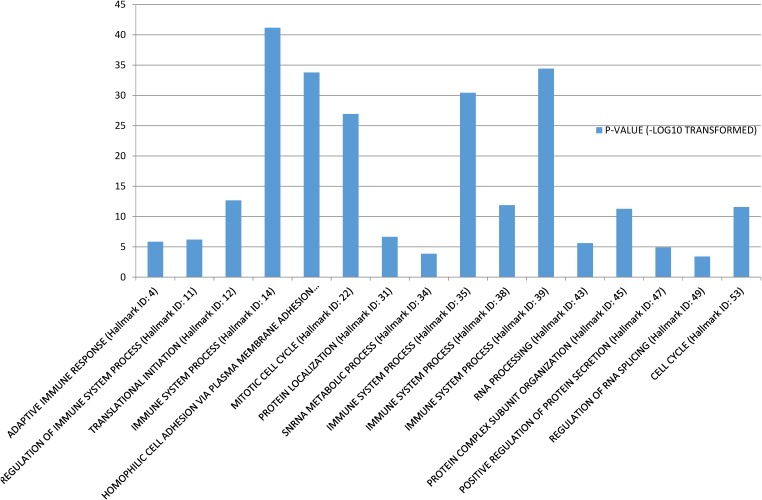
Function annotations of the conservative communities.

Gene Set Enrichment Analysis (Subramanian et al., 2005) was performed to investigate the biological functions of the 58 conserved communities. The function of each community was set as the most significant biological process. Among the 58 communities, 43 were significantly enriched. Some functional annotations of these communities are shown in Figure 1.

The enrichment of these communities mainly involved three types of biological processes. The first type was biological processes that were related to basic cellular functions, such as “Protein localization,” “RNA processing,” and “Regulation of RNA splicing.” The second type of biological process was related to immune system. There were four communities that were directly significantly related to “Immune system process,” and two were significantly related to “Adaptive immune response” Regulation of immune system process, respectively. As we know, avoiding immune destruction is one of the emerging hallmarks of cancer (Hanahan and Weinberg, 2011) and the immune response of cancer patients is essential for cancer therapy (Medler et al., 2015). Therefore, our findings concur with the findings of previous studies. The third type of biological process was related to the cell cycle and cell adhesion. For example, the 17th conserved community was significantly related to “Homophilic cell adhesion via plasma membrane adhesion molecules” and the 22nd and 53rd conserved communities were significantly related to “Mitotic cell cycle” and “Cell cycle,” respectively. Activating cancer cell invasion and metastasis is a cancer hallmark (Hanahan and Weinberg, 2011), and cell adhesion molecules are indispensable for cancer cell invasion and metastasis (Oka et al., 1993; Kannagi et al., 2004). Sustaining proliferative signaling is also a hallmark, and the cell cycle is an important component of these processes (Hanahan and Weinberg, 2011).

From the above results, a conclusion could be drawn that some conserved communities were indeed related to known cancer hallmarks.

### Two Cancer Hallmarks Can Distinguish the Prognostic Risks of Cancer Patients Regarding Four Types of Cancer

The genes involved in the conserved communities may be robust regarding accurate cancer prognosis. Therefore, we employed the genes involved in each conserved community as a prognostic signature to predict the prognostic risks of cancer patients in all the cancer data sets, so as to select the communities that could distinguish the prognostic risks of cancer patients regarding multiple cancers. These conserved communities that could distinguish the prognosis risks were regarded as cancer hallmarks.

As a result, many conserved communities were found to be able to distinguish the prognostic risks of cancer patients in at least one cancer data set (Supplementary Table S9). Among these hallmarks, two could predict the prognosis of cancer patients in four data sets. The number of LUAD sample was too small for survival analysis (Table 1), but the two hallmarks could distinguish between cancer patients in terms of survival in four out of the remaining six data sets.

**FIGURE 2:**
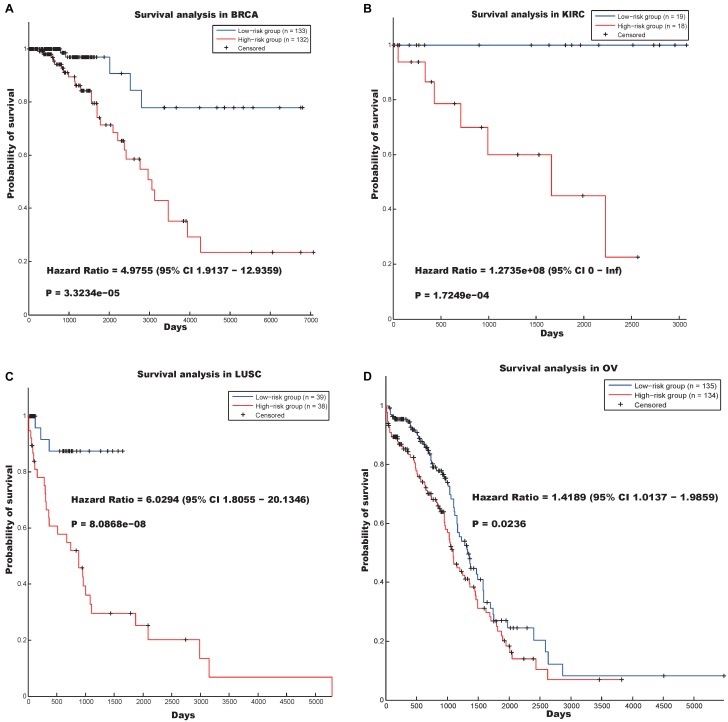
Survival analysis using the hallmark “Mitotic cell cycle” in four cancer data sets. **(A)** BRCA, **(B)** KIRC, **(C)** LUSC, and **(D)** OV.

The first hallmark was annotated as “Mitotic cell cycle” (*p*-value = 1.9200e-20), and the genes involved in this community (Supplementary Table S10) could distinguish between cancer patients in terms of survival in the BRCA, KIRC, LUSC, and OV data sets (Figure 2). Figure 2 indicates that the log-rank *p*-values of our prognostic model in the four data sets were 3.3234e-05, 1.7249e-04, 8.0868e-08, and 0.0236, respectively, and the hazard ratios in the four data sets were 4.9755, 1.2735e08, 6.0294, and 1.4189, respectively.

Obviously, deregulation of the cell cycle may result in aberrant cell proliferation, and the genes involved in the cell cycle may be appropriate biomarkers for cancer detection and prognosis (Williams and Stoeber, 2012). Our finding concurs with the findings of previous studies, which indicate that the genes involved in the “Mitotic cell cycle” community may be essential in the prognosis of multiple types of cancer.

**FIGURE 3:**
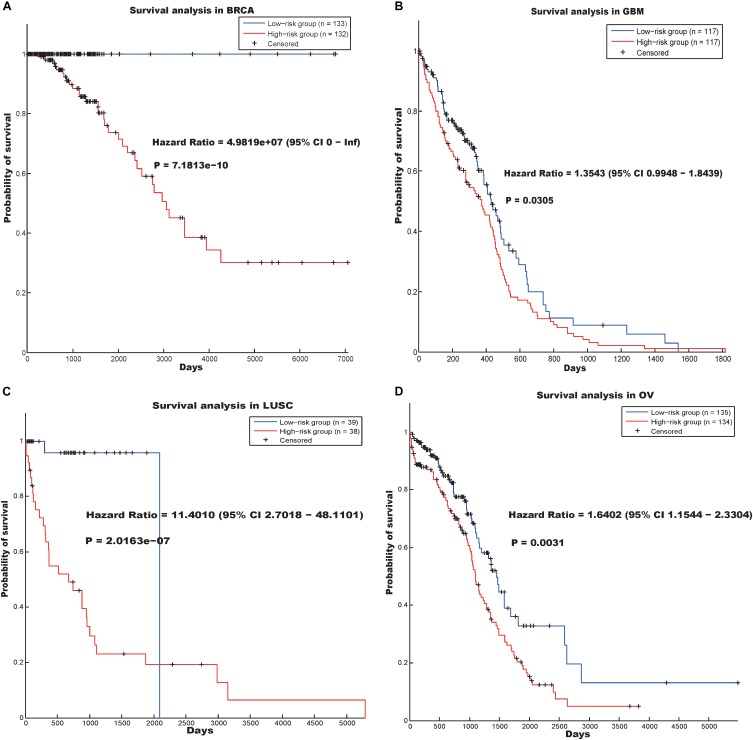
Survival analysis using the hallmark “RNA processing” in four cancer data sets. **(A)** BRCA, **(B)** GSM, **(C)** LUSC and (D) OV.

The other hallmark was annotated as “RNA processing” (*p*-value = 2.4000e-06). Survival analysis reflected that the prognostic model based on the genes involved in the “RNA processing” hallmark (Supplementary Table S11) could predict the prognosis of cancer patients in the BRCA, GBM, LUSC, and OV data sets (Figure 3).

In the BRCA data set, the hazard ratio between the high- and low-risk groups was 4.9819e07 and the log-rank *p*-value was 7.1813e-10. In addition, the hazard ratios of the patients divided by our prognostic model in the GBM, LUSC, and OV data sets were 1.3543 (*p*-value = 0.0305), 11.4010 (*p*-value = 2.0163e-07), and 1.6402 (*p*-value = 0.0031), respectively.

“RNA processing” is a biological process that is related to the basic function of the cell. Previous studies have reported that dysregulation of RNA processing may drive colorectal and lung cancer (Bordonaro, 2013; Sanidas et al., 2014), and RNA processing may be a potential therapeutic target in Ewing sarcoma (Grohar et al., 2016). Our results indicate that abnormal RNA processing may be common in cancers and useful in screening for cancer drugs.

### Cancer Hallmarks Could Be Useful for Screening for Cancer Drugs

As described above, two conserved communities could distinguish between cancer patients in terms of survival regarding four types of cancer, and the genes involved in the communities may be potential therapeutic targets. Therefore, these genes were used to screen for drugs.

We investigated 5837 drugs (along with drug targets and indications) from TTD (Chen et al., 2002), DGIdb (Wagner et al., 2016), and DrugBank (Wishart et al., 2008; Law et al., 2014). Each drug was identified as an appropriate drug if it could significantly target the genes in the either of the two hallmarks. Regarding the “Mitotic cell cycle” hallmark, 55 drugs were identified as appropriate drugs (Supplementary Table S12). Among these drugs, 48 (87%) were indicated for cancer. Of the 5837 drugs in the databases, 2442 (42%) were indicated for cancer. The proportion of drugs indicated for cancer that were identified using the hallmark was significantly higher, with a *p*-value of 2.7935e-12 based on the hypergeometric distribution test.

The genes involved in the “RNA processing” hallmark were also used to prioritize drugs. Of all the drugs tested, 57 were identified as appropriate drugs (Supplementary Table S13). Among these drugs, 31 (54%) were indicated for cancer. The hypergeometric distribution test indicated that the genes involved in the “RNA processing” hallmark could be used to screen for cancer drugs, with a *p*-value of 0.03712.

According to the above results, a conclusion could be drawn that the genes involved in the two hallmarks could be useful for screening for cancer drugs.

### Our Method Is Robust With Different Parameters

The hallmarks identified by our method may be influenced by the selection of different parameters or data sets. For example, the selection of different number of neighbors to construct the gene co-expression networks, and the using of cancer data sets for mining the communities. Therefore, we used different parameters and data sets to select the hallmarks and test whether the results are stable.

In this work, the rank-based method (Ruan et al., 2010) was used to construct the gene co-expression network for each cancer and top ten gene, which are the most relevant ones, were set as the neighbors for each gene. Here, when constructing the gene co-expression network, for each gene, we selected the top 5 genes as its neighbors. Beyond that, the same strategy was used to mine the conservative communities. As a result, 44 conservative communities were obtained and 23 unique GO terms was annotated (Supplementary Table S14). As we know, 37 unique GO terms were enriched by the original method. The number of common GO terms identified by the two methods was 7 and a *p*-value of 3.4379e-10 was obtained using the hypergeometric distribution test, based on the size of the universal set of 4436. In addition, the hallmark “Mitotic cell cycle” was also found in the intersection set of the two GO term sets.

In the original pipeline, as the number of samples with clinic information in OV data set was the largest, we mined the dense clusters based on the gene co-expression network of OV. To investigate whether the hallmarks would be biased by the selection of different data sets, we used the gene co-expression network of breast cancer (the number of samples of breast cancer in TCGA was also very big) to mine the communities, based on which, 57 conservative communities were obtained. Among the 57 conservative communities, 29 unique GO terms was annotated (Supplementary Table S15). The intersection set between the 29 different GO terms and the 37 unique GO terms identified by the original method was also significant, with the *p*-value of 6.1118e-13. What’s more, the “Mitotic cell cycle” was also enriched by this strategy.

**FIGURE 4:**
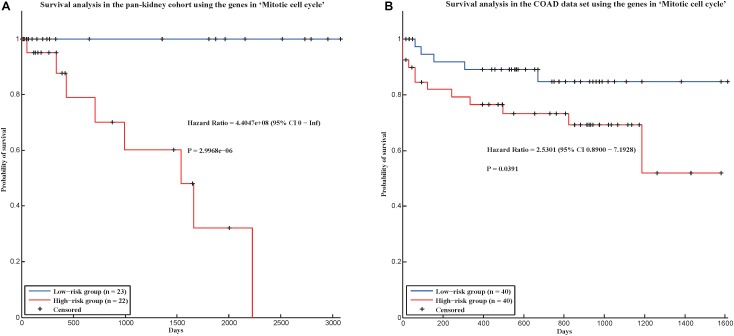
Survival analysis in two validation cohorts using the genes in “Mitotic cell cycle.” **(A)** Pan-kidney cohort and **(B)** COAD data set.

In addition, we applied our method in two new cancer data sets to evaluate whether our method could be applied in new data set. In TCGA, there was a pan-kidney cohort, which contained 88 samples from Kidney Chromophobe (KICH), KIRC, and Kidney renal papillary cell carcinoma (KIRP). Here, we also used the gene expression data of the pan-kidney cohort to construct a gene co-expression network, and test whether the 58 conservative communities identified by our method were also significant in this network. As a result, among the 58 conservative communities, 56 communities were significant (*p*-value < 0.05). Furthermore, we also used the genes in the hallmark “Mitotic cell cycle” (which could predict the prognostic risks of cancer patients in four cancers) to predict the prognostic risks of cancer patients in the pan-kidney cohort and found that the hallmark could also distinguish the prognostic risks of cancer patients in the data set (Figure 4A), with a log-rank *p*-value of 2.9968e-06. We also validate our hallmarks in the COAD data set, which contains gene expression profiles of 172 colon adenocarcinoma samples. As a result, all the 58 conservative communities existed in the gene co-expression network of COAD. In addition, the hallmark “Mitotic cell cycle” was also used to distinguish the prognostic risks of cancer patients in COAD. In this data set, the hazard ratio between the high-risk group and low-risk group is 2.5301 and *p*-value is 0.0391 (Figure 4B). That is, the genes in this hallmark could also be used to predict the prognosis of cancer patients in the COAD data set. Therefore, most of the conservative communities identified by our method were also conservative in the two new data sets and the genes in “Mitotic cell cycle” may be also important in the prognosis of the cancer patients of the two new data sets.

In conclusion, our method is robust with the selection of different parameters and could be applied in new data sets.

## Discussion

Although there is high heterogeneity among cancers, some hallmarks are still found. Most previous studies on cancer hallmarks mainly studied the biochemical/metabolic characteristics shared by several types of cancer. The identification of common genetic features is still a challenge. A community in a co-expression network for a particular cancer type may represent a functional unit in the formation or progression of cancer. The communities in the co-expression networks of multiple cancers may be common genetic features in cancers. In this study, using the gene expression data of seven cancers from TCGA, we used permutation tests to mine the conserved communities in the co-expression networks of these cancers in order to identify cancer hallmarks. First, the topological properties of all seven networks were shown to conform to the properties of typical biology networks. Based on the seven networks, 58 conserved communities were mined. Functional annotations of these communities showed that they were mainly related to basic cellular functions, cell cycle, and immune response. Most of the conserved communities could distinguish between cancer patients in terms of survival regarding at least one cancer. In particular, two hallmarks, which were related to “Mitotic cell cycle” and “RNA processing” could each predict the prognosis of cancer patients regarding four types of cancer. Furthermore, both hallmarks were useful for screening for cancer drugs. What’s more, we also evaluated the robustness of our method and found that some hallmarks could be identified by using different parameters or data sets and could be applied in new data sets.

Due to the high heterogeneity in cancers, it is hard to identify the driver genes. However, genes involved in the common genetic features of cancers may be more likely to be essential genes in cancer. The results indicate that these genes are indeed cancer related. In addition, these genes could be used to predict cancer prognosis and screen for cancer drugs. Our method may provide a new way to identify the key genes in cancer, and these genes may have clinical value.

In this study, we only mined cancer hallmarks using gene expression data. However, other genetic data and epigenetic data may also facilitate the identification of common genetic features of cancers. In the future, we will mine cancer hallmarks by integrating multi-omics data. Furthermore, experimental validation of the identified genes will be performed.

## Data Availability

The datasets generated for this study can be found in TCGA, https://confluence.broadinstitute.org/display/GDAC/Dashboard-Stddata.

## Author Contributions

X-HZ designed the research. X-HZ and L-HY performed the research and wrote the manuscript. L-HY and Q-WH analyzed the data. All authors revised the manuscript.

## Conflict of Interest Statement

The authors declare that the research was conducted in the absence of any commercial or financial relationships that could be construed as a potential conflict of interest.

## References

[B1] Aran D. Sirota M. Butte A. J. (2015). Systematic pan-cancer analysis of tumour purity. *Nat. Commun.* 6:8971. 10.1038/ncomms9971 26634437 PMC4671203

[B2] Bader G. D. Hogue C. W. V. (2003). An automated method for finding molecular complexes in large protein interaction networks. *BMC Bioinformatics* 4:2 10.1186/1471-2105-4-2 PMC149346 12525261

[B3] Benesty J. Chen J. Huang Y. Cohen I. (2009). *Pearson Correlation Coefficient.* Berlin: Springer 10.1007/978-3-642-00296-0_5

[B4] Bordonaro M. (2013). Crosstalk between Wnt signaling and RNA processing in colorectal cancer. *J. Cancer* 4 96–103. 10.7150/jca.5470 23386908 PMC3563071

[B5] Bredberg A. (2011). Cancer: more of polygenic disease and less of multiple mutations? A quantitative viewpoint. *Cancer* 117 440–445. 10.1002/cncr.25440 20862743

[B6] Chang H. Y. Nuyten D. S. A. Sneddon J. B. Hastie T. Tibshirani R. Sorlie T. (2005). Robustness, scalability, and integration of a wound-response gene expression signature in predicting breast cancer survival. *Proc. Natl. Acad. Sci. U.S.A.* 102 3738–3743. 10.1073/pnas.0409462102 15701700 PMC548329

[B7] Chen X. Guan N. N. Sun Y. Z. Li J. Q. Qu J. (2018a). MicroRNA-small molecule association identification: from experimental results to computational models. *Brief. Bioinform.* 20:bby098. 10.1093/bib/bby098 30325405

[B8] Chen X. Huang L. Xie D. Zhao Q. (2018b). EGBMMDA: extreme gradient boosting machine for MiRNA-disease association prediction. *Cell Death Dis.* 9:3. 10.1038/s41419-017-0003-x 29305594 PMC5849212

[B9] Chen X. Wang L. Qu J. Guan N. Li J. (2018c). Predicting miRNA-disease association based on inductive matrix completion. *Bioinformatics* 34 4256–4265. 10.1093/bioinformatics/bty503 29939227

[B10] Chen X. Xie D. Wang L. Zhao Q. You Z. Liu H. (2018d). BNPMDA: bipartite network projection for MiRNA–disease association prediction. *Bioinformatics* 34 3178–3186. 10.1093/bioinformatics/bty333 29701758

[B11] Chen X. Yin J. Qu J. Huang L. (2018e). MDHGI: matrix decomposition and heterogeneous graph inference for miRNA-disease association prediction. *PLoS Comput. Biol.* 14:e1006418. 10.1371/journal.pcbi.1006418 30142158 PMC6126877

[B12] Chen X. Huang L. (2017). LRSSLMDA: laplacian regularized sparse subspace learning for MiRNA-disease association prediction. *PLoS Comput. Biol.* 13:e1005912. 10.1371/journal.pcbi.1005912 29253885 PMC5749861

[B13] Chen X. Huang Y. You Z. Yan G. Wang X. (2016a). A novel approach based on KATZ measure to predict associations of human microbiota with non-infectious diseases. *Bioinformatics* 33 733–739. 10.1093/bioinformatics/btw715 28025197

[B14] Chen X. Ji Z. L. Chen Y. Z. (2002). TTD: therapeutic target database. *Nucleic Acids Res.* 30 412–415. 10.1093/nar/30.1.412 11752352 PMC99057

[B15] Chen X. Xie D. Zhao Q. You Z.-H. (2017). MicroRNAs and complex diseases: from experimental results to computational models. *Brief. Bioinform.* 19:bbx130. 10.1093/bib/bbx130 29045685

[B16] Chen X. Yan C. C. Zhang X. You Z. (2016b). Long non-coding RNAs and complex diseases: from experimental results to computational models. *Brief. Bioinform.* 18 558–576. 10.1093/bib/bbw060 27345524 PMC5862301

[B17] Dhawan A. Scott J. G. Harris A. L. Buffa F. M. (2017). Pan-cancer characterisation of microRNA with hallmarks of cancer reveals role of microRNA-mediated downregulation of tumour suppressor genes. *bioRxiv* [Preprint]. 10.1101/238675 PMC6286392 30531873

[B18] Domany E. (2014). Using high-throughput transcriptomic data for prognosis: a critical overview and perspectives. *Cancer Res.* 74 4612–4621. 10.1158/0008-5472.CAN-13-3338 25183786

[B19] Eytan D. (2014). Using high-throughput transcriptomic data for prognosis: a critical overview and perspectives. *Cancer Res.* 74 4612–4621. 10.1158/0008-5472.CAN-13-3338 25183786

[B20] Grohar P. J. Kim S. Rivera G. O. R. Sen N. Haddock S. Harlow M. (2016). Functional genomic screening reveals splicing of the EWS-FLI1 fusion transcript as a vulnerability in ewing sarcoma. *Cell Rep.* 14 598–610. 10.1016/j.celrep.2015.12.063 26776507 PMC4755295

[B21] Hanahan D. Weinberg R. A. (2011). Hallmarks of cancer: the next generation. *Cell* 144 646–674. 10.1016/j.cell.2011.02.013 21376230

[B22] Hu W. Zhou X. (2017). Identification of prognostic signature in cancer based on DNA methylation interaction network. *BMC Med. Genomics* 10:63. 10.1186/s12920-017-0307-9 29322932 PMC5763425

[B23] Jemal A. Murray T. Ward E. Samuels A. Tiwari R. C. Ghafoor A. (2010). Cancer statistics, 2005. *Cancer J. Clin.* 55 10–30. 10.3322/canjclin.55.1.10 15661684

[B24] Kannagi R. Izawa M. Koike T. Miyazaki K. Kimura N. (2004). Carbohydrate-mediated cell adhesion in cancer metastasis and angiogenesis. *Cancer Sci.* 95 377–384. 10.1111/j.1349-7006.2004.tb03219.x 15132763 PMC11159147

[B25] Knijnenburg T. A. Bismeijer T. Wessels L. F. Shmulevich I. (2015). A multilevel pan-cancer map links gene mutations to cancer hallmarks. *Chin. J. Cancer* 34 439–449. 10.1186/s40880-015-0050-6 26369414 PMC4593384

[B26] Law V. Knox C. Djoumbou Y. Jewison T. Guo A. C. Liu Y. (2014). DrugBank 4.0: shedding new light on drug metabolism. *Nucleic Acids Res.* 42 1091–1097. 10.1093/nar/gkt1068 24203711 PMC3965102

[B27] Mauger E. A. Wolfe R. A. Port F. K. (2010). Transient effects in the cox proportional hazards regression model. *Stat. Med.* 14 1553–1565. 10.1002/sim.4780141406 7481192

[B28] Mclendon R. Friedman A. Bigner D. Meir E. G. V. Van Meir E. Brat D. (2008). Comprehensive genomic characterization defines human glioblastoma genes and core pathways. *Nature* 455 1061–1068. 10.1038/nature07385 18772890 PMC2671642

[B29] Medler T. R. Cotechini T. Coussens L. M. (2015). Immune response to cancer therapy: mounting an effective antitumor response and mechanisms of resistance. *Trends Cancer* 1 66–75. 10.1016/j.trecan.2015.07.008 26457331 PMC4594836

[B30] Oka H. Shiozaki H. Kobayashi K. Inoue M. Tahara H. Kobayashi T. (1993). Expression of e-cadherin cell adhesion molecules in human breast cancer tissues and its relationship to metastasis. *Cancer Res.* 53 1696–1701. 8453644

[B31] Ruan J. Dean A. K. Zhang W. (2010). A general co-expression network-based approach to gene expression analysis: comparison and applications. *BMC Syst. Biol.* 4:8. 10.1186/1752-0509-4-8 20122284 PMC2829495

[B32] Sanidas I. Polytarchou C. Hatziapostolou M. Ezell S. A. Kottakis F. Hu L. (2014). Phosphoproteomics screen reveals Akt isoform-specific signals linking RNA processing to lung cancer. *Mol. Cell.* 53 577–590. 10.1016/j.molcel.2013.12.018 24462114 PMC3947584

[B33] Sotiriou C. Wirapati P. Loi S. Harris A. L. Fox S. B. Smeds J. (2006). Gene expression profiling in breast cancer: understanding the molecular basis of histologic grade to improve prognosis. *J. Natl. Cancer Inst.* 98 262–272. 10.1093/jnci/djj052 16478745

[B34] Stewart B. W. Freddie B. David F. Hiroko O. Kurt S. Andreas U. (2016). Cancer prevention as part of precision medicine: ‘plenty to be done’. *Carcinogenesis* 37 2–9. 10.1093/carcin/bgv166 26590901 PMC4700936

[B35] Subramanian A. Tamayo P. Mootha V. K. Mukherjee S. Ebert B. L. Gillette M. A. (2005). Gene set enrichment analysis: a knowledge-based approach for interpreting genome-wide expression profiles. *Proc. Natl. Acad. Sci. U.S.A.* 102 15545–15550. 10.1073/pnas.0506580102 16199517 PMC1239896

[B36] Wagner A. H. Coffman A. C. Ainscough B. J. Spies N. C. Skidmore Z. L. Campbell K. M. (2016). DGIdb 2.0: mining clinically relevant drug-gene interactions. *Nucleic Acids Res.* 44 D1036–D1044. 10.1093/nar/gkv1165 26531824 PMC4702839

[B37] Williams G. H. Stoeber K. (2012). The cell cycle and cancer. *J. Pathol.* 226 352–364. 10.1002/path.3022 21990031

[B38] Wishart D. S. Knox C. Guo A. C. Cheng D. Shrivastava S. Dan T. (2008). DrugBank: a knowledgebase for drugs, drug actions and drug targets. *Nucleic Acids Res.* 36 901–906. 10.1093/nar/gkm958 18048412 PMC2238889

[B39] Yang Y. Han L. Yuan Y. Li J. Hei N. Liang H. (2014). Gene co-expression network analysis reveals common system-level properties of prognostic genes across cancer types. *Nat. Commun.* 5:3231. 10.1038/ncomms4231 24488081 PMC3951205

[B40] You Z. Huang Z. Zhu Z. Yan G. Li Z. Wen Z. (2017). PBMDA: a novel and effective path-based computational model for miRNA-disease association prediction. *PLoS Comput. Biol.* 13:e1005455. 10.1371/journal.pcbi.1005455 28339468 PMC5384769

[B41] Zhang W. Chen Y. Li D. Yue X. (2018a). Manifold regularized matrix factorization for drug-drug interaction prediction. *J. Biomed. Inform.* 88 90–97. 10.1016/j.jbi.2018.11.005 30445219

[B42] Zhang W. Liu X. Chen Y. Wu W. Wang W. Li X. (2018b). Feature-derived graph regularized matrix factorization for predicting drug side effects. *Neurocomputing* 287 154–162. 10.1016/j.neucom.2018.01.085

[B43] Zhang W. Qu Q. Zhang Y. Wang W. (2018c). The linear neighborhood propagation method for predicting long non-coding RNA–protein interactions. *Neurocomputing* 273 526–534. 10.1016/j.neucom.2017.07.065

[B44] Zhang W. Yue X. Huang F. Liu R. Chen Y. Ruan C. (2018d). Predicting drug-disease associations and their therapeutic function based on the drug-disease association bipartite network. *Methods* 145 51–59. 10.1016/j.ymeth.2018.06.001 29879508

[B45] Zhang W. Yue X. Lin W. Wu W. Liu R. Huang F. (2018e). Predicting drug-disease associations by using similarity constrained matrix factorization. *BMC Bioinformatics* 19:223. 10.1186/s12859-018-2220-4 29914348 PMC6006580

[B46] Zhang W. Yue X. Tang G. Wu W. Huang F. Zhang X. (2018f). SFPEL-LPI: sequence-based feature projection ensemble learning for predicting LncRNA-protein interactions. *PLoS Comput. Biol.* 14:e1006616. 10.1371/journal.pcbi.1006616 30533006 PMC6331124

[B47] Zhang W. Chen Y. Liu F. Luo F. Tian G. Li X. (2017a). Predicting potential drug-drug interactions by integrating chemical, biological, phenotypic and network data. *BMC Bioinformatics* 18:18. 10.1186/s12859-016-1415-9 28056782 PMC5217341

[B48] Zhang W. Yue X. Liu F. Chen Y. Tu S. Zhang X. (2017b). A unified frame of predicting side effects of drugs by using linear neighborhood similarity. *BMC Syst. Biol.* 11:101. 10.1186/s12918-017-0477-2 29297371 PMC5751767

[B49] Zhou X. Liu J. Wang W. (2014). Construction and investigation of breast-cancer-specific ceRNA network based on the mRNA and miRNA expression data. *IET Syst. Biol.* 8 96–103. 10.1049/iet-syb.2013.0025 25014376 PMC8687191

